# AexB is an aromatic amino acid exporter that functions as a metabolic safety valve

**DOI:** 10.1128/mbio.00231-26

**Published:** 2026-04-03

**Authors:** Blake A. Everett, Arthur Prindle

**Affiliations:** 1 Department of Biochemistry and Molecular Genetics, Feinberg School of Medicine, Chicago, Illinois, USA; 2 Center for Synthetic Biology, Northwestern University 3270, Evanston, Illinois, USA; 3 Department of Chemical and Biological Engineering, Northwestern University 3270, Evanston, Illinois, USA; 4 Biohub, Chicago, Illinois, USA; The University of Texas Health Science Center at Houston John P. and Katherine G. McGovern Medical School, Houston, Texas, USA

**Keywords:** amino acids, transporters, metabolism, bacteria, *Bacillus subtilis*

## Abstract

Aromatic amino acids—tryptophan, tyrosine, phenylalanine, and histidine—are essential for bacterial growth and are among the most energetically expensive metabolites to synthesize. Despite this cost, it has been recently shown that bacteria possess exporters for these amino acids. Here, we identify *aexB* (formerly *yvjA*) as a gene encoding a novel aromatic amino acid exporter in *Bacillus subtilis*. Using a transposon-based screen, we found that *aexB* overexpression confers resistance to the toxic tryptophan analog 5-fluorotryptophan. Additional analog screens revealed that AexB also promotes tolerance to toxic derivatives of tyrosine, phenylalanine, and histidine but not non-aromatic amino acids. LC-MS analysis showed that AexB specifically exports aromatic amino acids, and co-culture assays confirmed that overexpression of *aexB* can support the growth of aromatic amino acid auxotrophs. Furthermore, overexpression of *aexB* impaired growth when intracellular tryptophan was limiting. On the other hand, deletion of *aexB* exacerbated growth defects under excess tryptophan conditions, likely due to feedback inhibition of aromatic amino acid synthesis pathways. Our findings reveal that AexB is an aromatic amino acid exporter that functions as a metabolic safety valve.

**IMPORTANCE:**

Identification and characterization of amino acid exporters is a broadly relevant topic. Amino acid synthesis is energetically costly, and thus functional relevance for their export is unintuitive. Identification of the molecular components that allow export may offer new engineering opportunities to improve biomanufacturing and metabolic engineering. Characterization of these exporters may also provide a more complete understanding of the human microbiome where amino acids, especially tryptophan, have been established as nodes of crosstalk between host and microbiota.

## INTRODUCTION

Amino acids are essential building blocks of proteins and are thus imperative to life. There has been considerable effort to study amino acid import, metabolism, and incorporation into proteins; however, until recently, there has been little investigation into their export. Since the 1950s, when it was discovered that *Corynebacterium glutamicum* efficiently secretes glutamate, amino acid secretion has been an important topic in biotechnological research. However, until the 1990s, secretion of amino acids was largely assumed to be a passive, cell wall diffusion-mediated process which only occurred under artificial industrial processes (1). Finally, in 1996, LysE became the first identified bacterial amino acid exporter (2). Since the identification of LysE, many amino acid exporters have been identified in bacteria (3–20). It has been proposed that these exporters function to maintain homeostasis, as has been observed for other metabolites in bacteria (21–24), and/or to export deleterious molecules (10, 14, 25–27). It is well appreciated that d-amino acids can interfere with translation, amino acid synthesis, and other cellular processes (28–31). Still, canonical l-amino acids can be growth inhibitory. This can occur via feedback inhibition in overlapping synthesis pathways, but can also be due to the accumulation of toxic intermediates or products of side reactions when homeostasis is disrupted (32–34). Aromatic amino acids specifically are interesting in this context, as their biosynthesis is particularly expensive, with *de novo* tryptophan, phenylalanine, and tyrosine synthesis requiring approximately 74, 52, and 50 ATP equivalents of energy per molecule, respectively (35). While one bacterial aromatic amino acid exporter has been identified (13), due to this high energetic investment, it remains unclear what benefit their secretion serves.

Two amino acid exporters have recently been identified in *B. subtilis*. AzlCD has been shown to export asparagine, histidine, and 4-azaleucine (6, 25, 36) and AexA has been shown to export C3-amino acids, including alanine, serine, and 2,3-diaminopropionic acid, as well as glycine (10). This raises the question of how prevalent these amino acid exporters are in *B. subtilis* as well as other bacteria. *B. subtilis* is naturally found in the rhizosphere, the region of soil surrounding plant roots. Interestingly, it has been shown that tryptophan levels are significantly enriched in the rhizosphere compared to bulk soil (37). Given the broad relevance of tryptophan and the relatively high levels of tryptophan in their native environment, we wondered if *B. subtilis* may encode uncharacterized tryptophan exporters.

**Fig 1:**
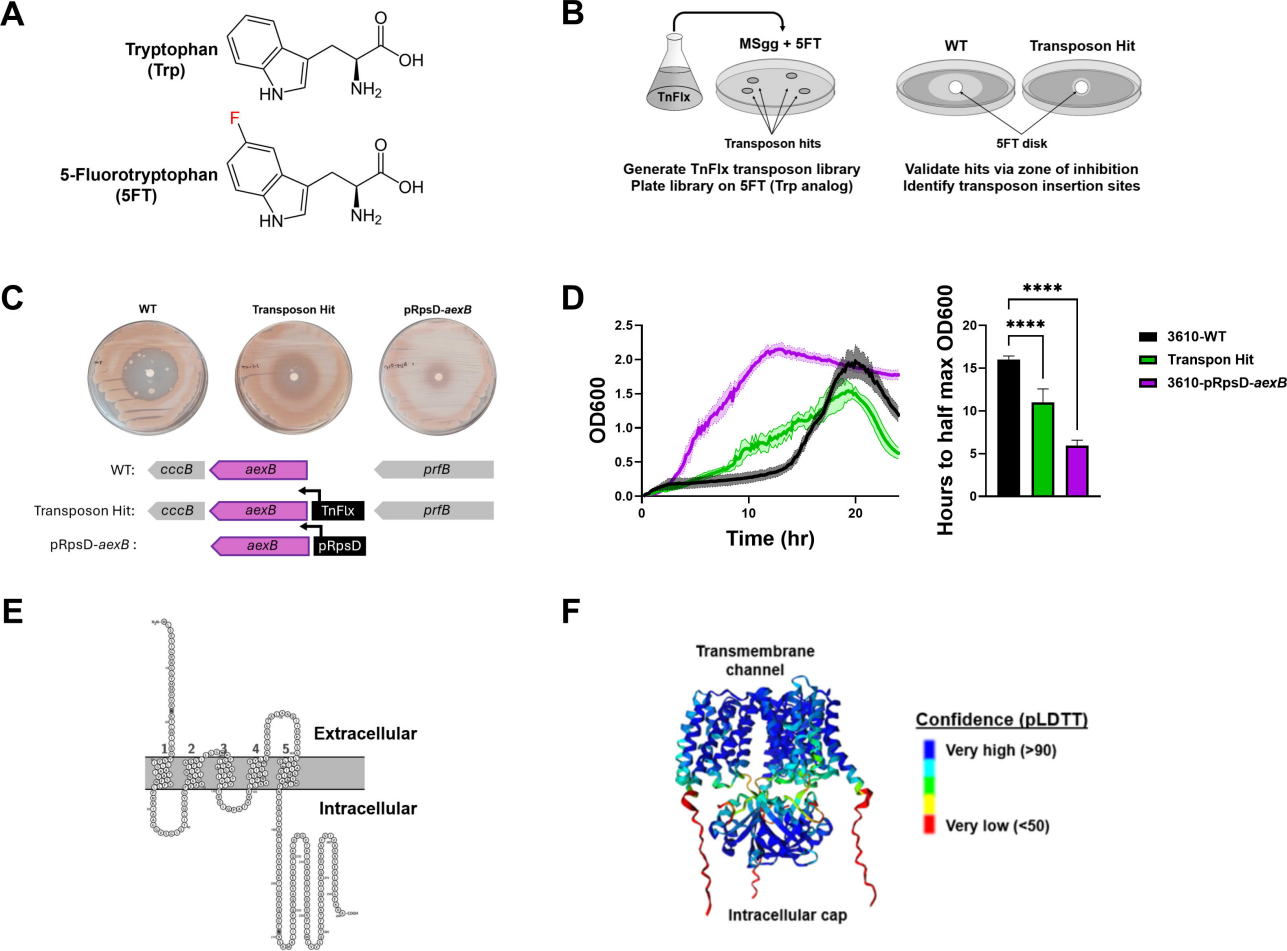
Transposon screen identifies overexpression of uncharacterized gene, *aexB*, allows for increased survival on 5FT. (**A**) Structures of tryptophan and 5-fluorotryptophan. (**B**) Schematic depicting the transposon-based screen to identify mutants which can grow on levels of 5FT that are toxic to WT. (**C**) Representative images from zone of inhibition experiment using filter disks soaked in 50 µg of 5FT, and the corresponding genetic contexts. (**D**) Average growth curves in 96-well plate reader format for 3610-WT, a representative transposon hit, and 3610-pRpsD-*aexB* in MSgg supplemented with 0.4 mM 5-FT (left) and quantification of the time it took for each strain to reach half the maximum OD_600_ (right). Shaded area is SEM. Statistical significance was determined by one-way ANOVA followed by *post hoc* multiple comparisons. *n* = 12, ****P < 0.0001. (**E**) Protter predicted transmembrane domains for an AexB monomer. (**F**) AlphaFold predicted structure for an AexB trimer.

In *B. subtilis*, intracellular tryptophan levels are regulated by the gene product of *mtrB*, the tryptophan attenuation protein (TRAP). The TRAP undecamer binds up to 11 tryptophan molecules and in this active conformation acts as a transcriptional and translational repressor by binding the nascent mRNAs for tryptophan-related genes. The 5′ segment of the tryptophan biosynthesis transcript (*trpEDCFBA*) contains mutually exclusive, overlapping antiterminator and terminator sequences. The TRAP binding site also overlaps with the antiterminator sequence, and bound TRAP allows formation of the terminator structure and prevents further transcription. TRAP also functions as a translational repressor for the tryptophan biosynthesis genes *trpE*, *trpD*, and *trpG*, as well as the only known tryptophan transporter, *trpP*, and an uncharacterized gene *ycbK*. This occurs via TRAP binding to sequences overlapping in the mRNA Shine-Dalgarno sequences*,* occluding the ribosome (38). *trpP* was first identified due to the presence of the TRAP binding motif and evidence for its transport capability was gained through use of the toxic tryptophan analog, 5-fluorotryptophan (5FT). 5FT has large structural overlap with tryptophan, the only difference being the addition of a fluorine atom to the 5-carbon of the benzene ring (Fig. 1A). This toxic analog functions through competition with tryptophan during tRNA charging, ultimately resulting in its incorporation into proteins, which have abrogated function (39–41). As such, it is not surprising that a tryptophan transporter would have affinity for 5FT. This was precisely the case, as it was shown that deletion of *trpP* led to increased resistance to 5FT (39). Given the high energetic cost of tryptophan synthesis, the recent discovery of amino acid exporters in *B. subtilis*, and the precedent of using 5FT to establish tryptophan transport activity of TrpP, we sought to identify tryptophan exporters in *B. subtilis*.

To identify tryptophan exporters, we made use of a genome-saturating, promoter-based, overexpression transposon library to screen for strains that are resistant to the toxic tryptophan analog 5FT. This screen identified that overexpression of the uncharacterized gene *yvjA* resulted in resistance to 5FT. Further characterization of YvjA revealed that it is an aromatic amino acid exporter, prompting renaming of the gene to *aexB* following the nomenclature for a recently characterized amino acid exporter in *B. subtilis,* AexA (10). We show that AexB is capable of exporting tryptophan, tyrosine, phenylalanine, and histidine, functioning as a metabolic safety valve.

## RESULTS

### Transposon screen for tryptophan exporters identifies *aexB*

To screen for tryptophan exporters, we utilized the *mariner*-based transposon, TnFlx, in *B. subtilis* NCIB3610 (3610-WT). This transposon utilizes an outward-facing, IPTG-inducible promoter, pHyperspank, and inserts at two-base pair “TA” sequences throughout the genome (42). Therefore, transposon mutagenesis results in not only a comprehensive knockout library, as insertion into open reading frames (ORFs) will cause disruption of that gene, but also a comprehensive overexpression library, as insertion upstream of an ORF will cause overexpression of that gene. The resulting library was then plated on toxic levels of 5FT, and resulting colonies were rescreened via a zone of inhibition (ZOI) assay with filter disks containing 50 µg of 5FT. Transposon hits that passed the ZOI assay were sent for whole-genome sequencing to identify the site of transposon insertion (Fig. 1B). In total, 43 colonies were sequenced, 27 of which had insertions 33 bp upstream of the uncharacterized gene *aexB* (Fig. S1).

To verify the 5FT resistance phenotype was due to overexpression of *aexB*, we constructed an overexpression strain in the 3610-WT background by overexpressing *aexB* in the ectopic locus, *amyE*, driven by the constitutive promoter, pRpsD (3610-pRpsD-*aexB*). 3610-pRpsD-*aexB* did have a reduced 5FT ZOI compared to wild type (WT) (Fig. 1C). Further, 3610-pRpsD-*aexB* grew equivalently to WT in the liquid defined minimal media, MSgg (Fig. S2), but had significantly improved growth compared to WT in MSgg with 0.4 mM 5FT (Fig. 1D). To gain insights into the possible function of AexB, we pursued structural predictions using Protter and AlphaFold. Protter predicts an AexB monomer to have five transmembrane domains and a large intracellular component with similarity to PII-sensing domains (Fig. 1E). Modeling trimers of AexB in AlphaFold predicts a large transmembrane channel with PII-sensing domains forming a cap-like structure for the channel (Fig. 1F). This suggested AexB was identified in the transposon screen because overexpression allows increased export of 5FT. Due to the high similarity between 5FT and tryptophan, we hypothesized that AexB is a tryptophan exporter.

### AexB appears to export tryptophan

**Fig 2:**
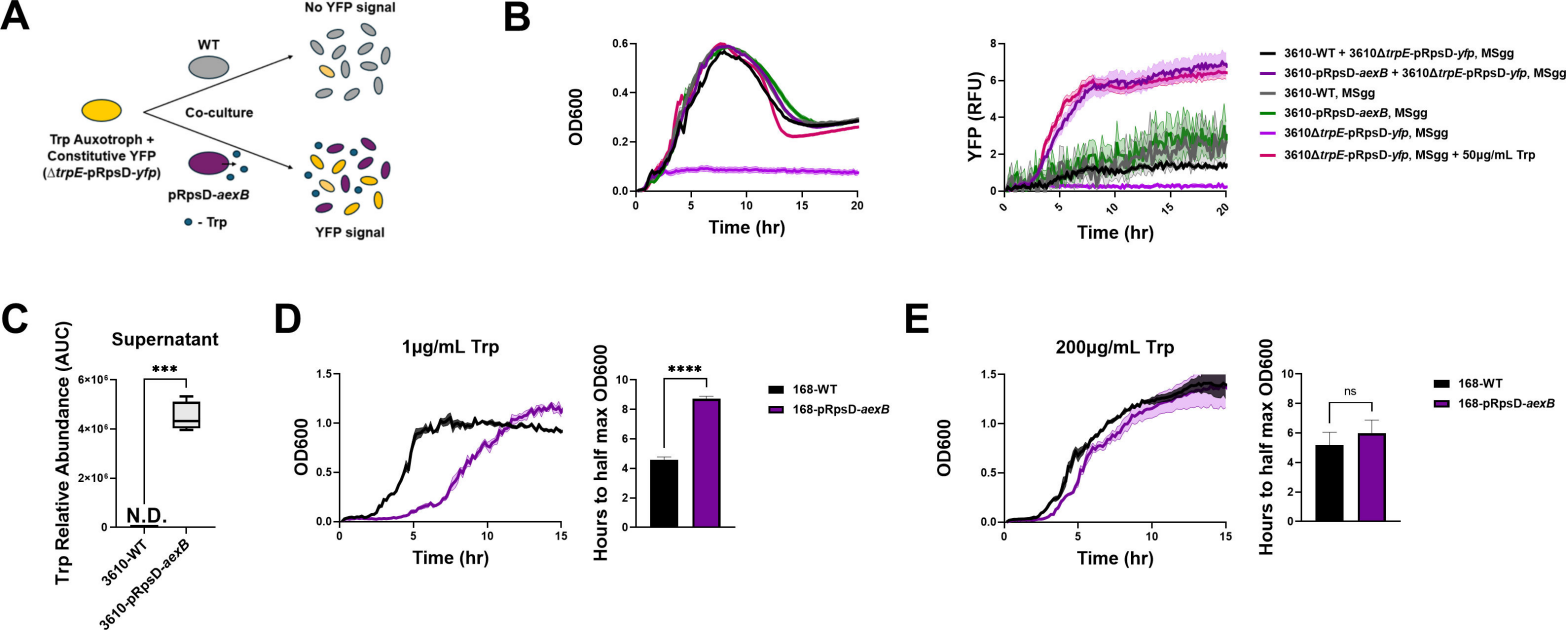
AexB is a tryptophan exporter. (**A**) Schematic depicting the experimental design for the co-culture experiment in panel B. (**B**) Average growth curves (left) and YFP signal (right) in 96-well plate reader format for a tryptophan auxotroph expressing a constitutive YFP reporter, 3610∆*trpE*-pRpsD-*yfp*, co-cultured with either 3610-WT or 3610-pRpsD-*aexB* in MSgg. Single-strain growth curves and YFP signals are also shown for all three strains, as well as 3610∆*trpE*-pRpsD-*yfp* grown in MSgg supplemented with 50 µg/mL Trp. Shaded area is SEM (*n* = 12). (**C**) LC-MS quantification of tryptophan in supernatants of 3610-WT and 3610-pRpsD-*aexB* MSgg liquid cultures. Statistical significance was determined by a Student’s *t*-test (*n* = 4, ***P = 0.0006) (**D**) Average growth curves for the tryptophan auxotrophic strain 168, in MSgg with low Trp (1 µg/mL) (left) and the corresponding average time to half max OD_600_ (right). Shaded area is SEM. Statistical significance was determined by one-way ANOVA followed by *post hoc* multiple comparisons (*n* = 4, ****P < 0.0001). (**E**) Same as panel D but with high Trp (200 µg/mL) (*n* = 4).

To test the hypothesis that AexB exports tryptophan, we pursued a co-culture experiment with a tryptophan auxotroph, 3610∆*trpE* (Fig. 2A). TrpE encodes anthranilate synthase, the first enzyme in the tryptophan biosynthetic pathway. As such, deletion of *trpE* confers tryptophan auxotrophy. In brief, if 3610-pRpsD-*aexB* is exporting tryptophan, co-culture with 3610∆*trpE* should be sufficient to support 3610∆*trpE* growth, while a co-culture of 3610-WT and 3610∆*trpE* should not support 3610∆*trpE* growth. Since OD cannot distinguish between the growth of the two strains in culture, we expressed YFP driven by pRpsD in the ectopic locus, *amyE,* in 3610∆*trpE*, such that growth could be determined. The results supported the hypothesis that 3610-pRpsD-*aexB* is exporting tryptophan, as the YFP signal increased over time in the co-culture of 3610-pRpsD-*aexB* and 3610∆*trpE* but not 3610-WT and 3610∆*trpE* (Fig. 2B). To approximate the amount of tryptophan that is exported by 3610-pRpsD-*aexB* over the course of its growth, we compared fluorescence in co-culture with 3610∆*trpE* to fluorescence of 3610∆*trpE* alone in varying levels of tryptophan. We estimate AexB overexpression results in between 0.1 and 1 µg/mL of tryptophan (~0.5–5 µM) (Fig. S3). These results suggest that AexB is a tryptophan exporter.

To corroborate these findings, we measured the levels of tryptophan in supernatant from 3610-WT and 3610-pRpsD-*aexB* cultures using LC-MS. Cultures were grown for 10 h in liquid MSgg and harvested by centrifugation allowing separation of cellular material and supernatant. While tryptophan was not detectable in the 3610-WT supernatant, an average of 4.48 × 10^6^ AU of tryptophan was detected in the 3610-pRpsD-*aexB* supernatants (Fig. 2C). This finding supports that AexB is a tryptophan exporter.

To gain further evidence that AexB is a tryptophan exporter, we investigated whether overexpression of AexB in low tryptophan conditions could be detrimental to growth. If AexB is indeed an exporter, overexpression when tryptophan is limiting should inhibit growth compared to WT. To test this hypothesis, we used *B. subtilis* 168, a closely related strain that is naturally auxotrophic for tryptophan due to a mutation in *trpC* (43). Both 3610 and 168 are derived from the Marbug lineage of *B. subtilis*. 168 is considered a laboratory “domesticated” strain, while 3610 more closely reflects wild-type strains. The two have a high degree of genetic overlap, with the major difference being the absence of an ~84 kb extra-chromosomal plasmid in 168, as well as a few other chromosomal mutations such as the aforementioned inactivation of *trpC* (44). Further, our growth experiments revealed that 168 appears to have reduced tryptophan uptake compared to 3610. Deletion of the only characterized tryptophan importer, *trpP*, in 168 caused a growth defect in MSgg supplemented with 50 µg/mL tryptophan compared to WT, while no such growth defect was observed in a 3610 tryptophan auxotroph (Fig. S4). This suggests that 168 has a lower tryptophan transport capacity than 3610 and ultimately lower intracellular tryptophan content when tryptophan is limiting. Leveraging this, we were able to show that overexpression of AexB causes a growth defect in 168 at low tryptophan (1 µg/mL) (Fig. 2D) but not at high tryptophan (200 µg/mL) (Fig. 2E). Taken together, these data confirm that AexB is a tryptophan exporter.

### AexB appears to export aromatic amino acids

**Fig 3:**
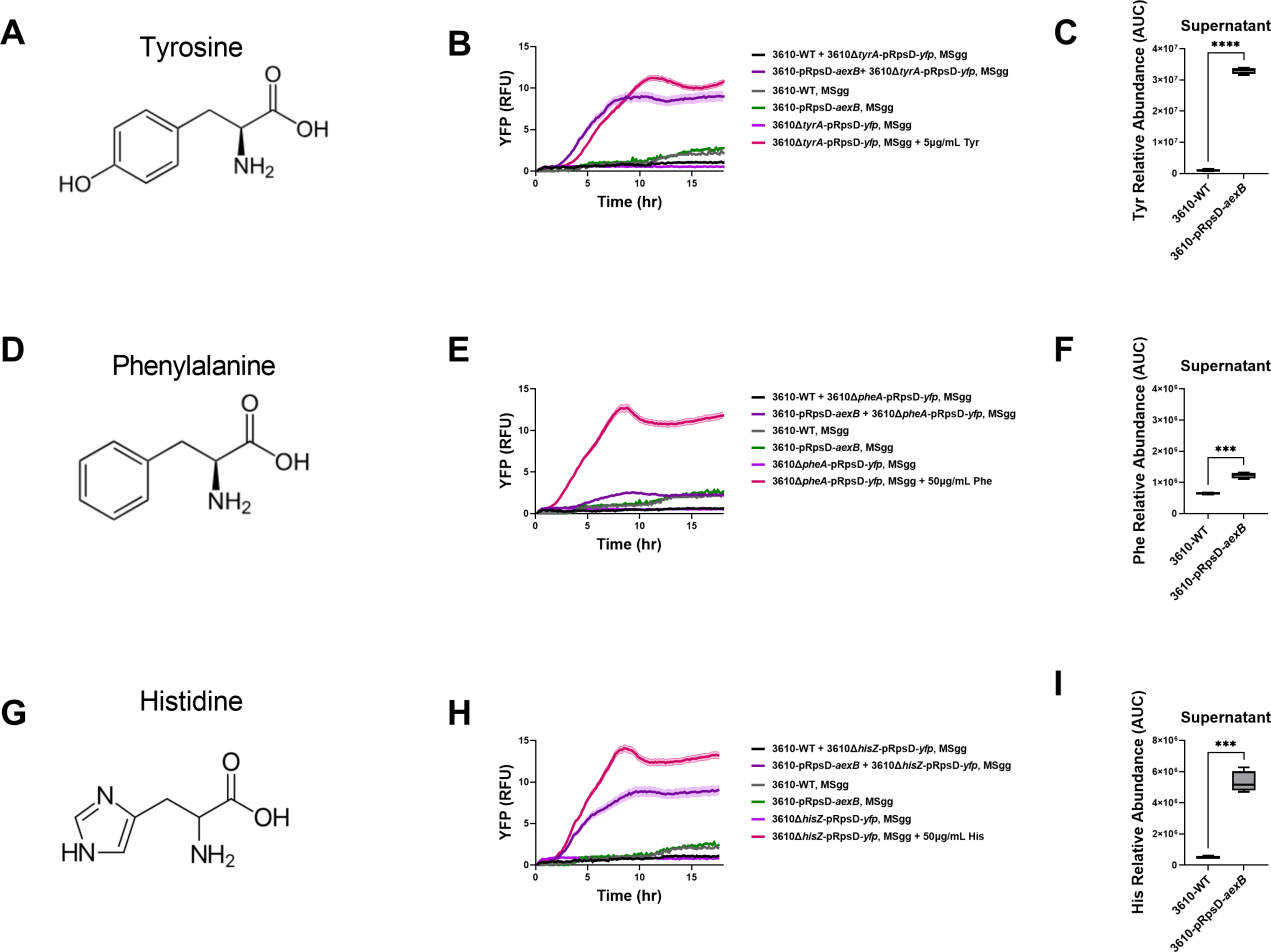
AexB exports aromatic amino acids. (**A**) Structure of tyrosine. (**B**) Same experimental design as in Fig. 2A and B, but using a tyrosine auxotroph expressing a constitutive YFP reporter, 3610∆*tyrA*-pRpsD-*yfp*. Shaded area is SEM (*n* = 12). (**C**) LC-MS quantification of tyrosine in the supernatants of 3610-WT and 3610-pRpsD-*aexB* MSgg liquid cultures. Statistical significance was determined by a student’s *t*-test (*n* = 4, ****P < 0.0001). (**D–F**) Same as panels **A–C**, but for phenylalanine instead of tyrosine (***P = 0.0007). (**G–I**) Same as panels **A–C**, but for histidine instead of tyrosine (***P = 0.0006).

We wondered if AexB could export other amino acids given that previously characterized amino acid exporters are promiscuous and that AexB appears to export both tryptophan and the tryptophan analog, 5FT. We examined the growth of 3610-pRpsD-*aexB* on a wide variety of amino acid analogs. If overexpression of AexB supported growth on these inhibitory analogs, this could be evidence that the cognate amino acids are substrates of AexB. We found that 3610-pRpsD-*aexB* had improved growth compared to 3610-WT in MSgg supplemented with meta-tyrosine or 4-fluorophenylalanine but none of the non-aromatic amino acid analogs tested (Fig. S5). This suggested that AexB was able to transport aromatic amino acids. To further investigate this, we repeated co-culture experiments but instead used tyrosine, phenylalanine, and histidine auxotrophic strains. The tyrosine auxotroph was able to grow in MSgg when co-cultured with 3610-pRpsD-*aexB* but not 3610-WT, as evidenced by increasing YFP signal (Fig. 3B). Furthermore, 3610-pRpsD-*aexB* supernatant had significantly higher levels of tyrosine than 3610-WT as measured by LC-MS (Fig. 3C). Similar phenotypes were observed for histidine (Fig. 3H and I). Taken together, this strongly suggests that AexB can export tyrosine and histidine. 3610-pRpsD-*aexB* was unable to support growth of the phenylalanine auxotroph (Fig. 3E). However, phenylalanine was significantly higher in the supernatant of 3610-pRpsD-*aexB* than 3610-WT (Fig. 3F). This may suggest that AexB can export phenylalanine, but with a lower affinity than tryptophan, tyrosine, and histidine.

### Export of aromatic amino acids via AexB maintains metabolic homeostasis

**Fig 4:**
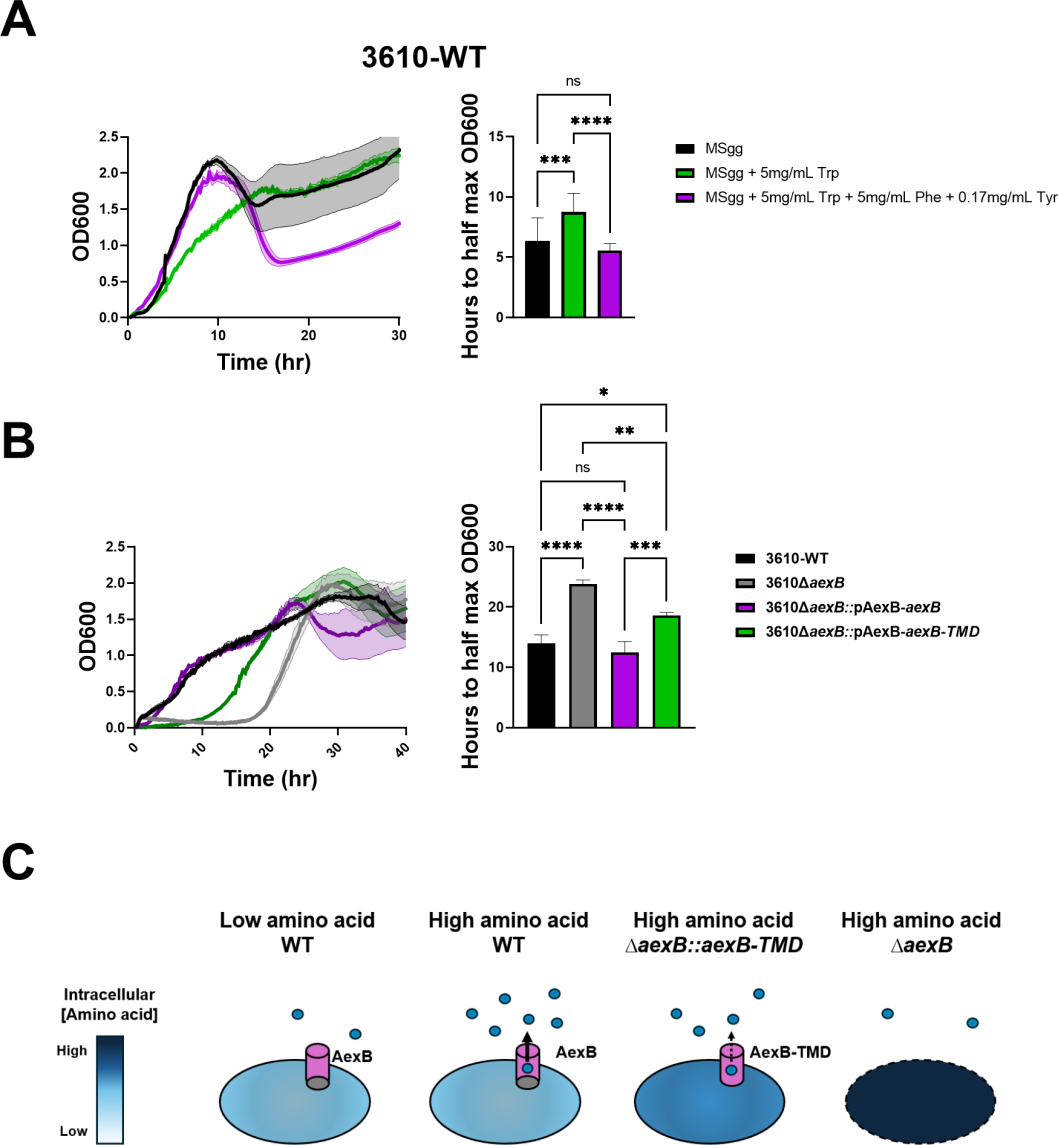
AexB maintains tryptophan homeostasis. (**A**) Average growth curves in 96-well plate reader format for 3610-WT in MSgg with or without trp alone or trp, phe, and tyr (left) and quantification of the average time it took to reach half the maximum OD_600_ in each condition (right). Shaded area is SEM. Statistical significance was determined by one-way ANOVA followed by *post hoc* multiple comparisons (*n* = 12, ***P = 0.0009 and ****P < 0.0001). (**B**) Average growth curves in 96-well plate reader format for 3610-WT, 3610∆*aexB,* 3610∆*aexB::*pAexB-*aexB* and 3610∆*aexB::*pAexB-*aexB-TMD* in MSgg + 8 mg/mL trp (left) and quantification of the average time it took to reach half the maximum OD_600_ for each strain (right). Shaded area is SEM. Statistical significance was determined by one-way ANOVA followed by post hoc multiple comparisons (*n* = 12, *P = 0.0106, **P = 0.0022, ***P = 0.0001, and ****P < 0.0001). (**C**) Model for how AexB functions as an aromatic amino acid safety valve.

It has previously been shown that in cyanobacteria, exogenous phenylalanine can become growth inhibitory due to feedback inhibition of enzymes in aromatic amino acid biosynthesis (34). We wondered if *B. subtilis* might have similar feedback mechanisms for tryptophan. Indeed, we observed that at concentrations of, or above, 5 mg/mL tryptophan 3610-WT has a growth defect (Fig. S6). We found that tryptophan growth inhibition could be reversed by the addition of phenylalanine and tyrosine to the growth medium (Fig. 4A). This suggests that phenylalanine and tyrosine synthesis were reduced by feedback inhibition in the presence of excess intracellular tryptophan. This led us to the hypothesis that AexB acts to export tryptophan, as well as the other aromatic amino acids, to prevent metabolic backup and allow continued aromatic amino acid synthesis. Consistent with this, we found that the tryptophan-induced growth defect is exacerbated by deletion of *aexB* and can be rescued by complementation (Fig. 4B). Taken together, we propose a model in which AexB activity is most relevant at high aromatic amino acid concentrations and functions as a metabolic safety valve (Fig. 4C).

Based on the AlphaFold predicted trimer structure, we hypothesized the cap domain of AexB may interact with tryptophan and increase export efficiency through the transmembrane domain. To assess the transport contribution of the cap domain, we constructed a capless, transmembrane domain-only version of AexB, 3610∆*aexB*::pAexB-*aexB-*TMD. Deletion of the cytoplasmic cap domain resulted in a growth defect in high tryptophan compared to the full-length transporter, but less severe compared to *aexB* deletion, supporting our hypothesis (Fig. 4B). We confirmed that these growth defects are specific to high tryptophan conditions, as these strains grow equivalently in unaltered MSgg (Fig. S7). Consistent with this, overexpression of the transmembrane domain-only version of AexB did not result in a growth defect at low tryptophan (Fig. S8). This suggests the cap contributes to efficient export of tryptophan through the transmembrane channel (Fig. 4C). Our results identify AexB as an aromatic amino acid exporter and indicate a function in maintaining homeostasis by acting as a metabolic safety valve to allow continued amino acid synthesis in the presence of high environmental concentrations.

## DISCUSSION

The results presented in this study identify the previously uncharacterized gene, *aexB*, as an aromatic amino acid exporter. Specifically, AexB appears to export tryptophan, tyrosine, phenylalanine, and histidine, as well as the amino acid analogs, 5FT, meta-tyrosine, and 4-fluorophenylalanine. While AexB did not appear to export any non-aromatic amino acids tested, other substrates may still be identified upon further testing. We also show that when concentrations of tryptophan reach or exceed 5 mg/mL, *B. subtilis* has a growth defect and this growth inhibition can be overcome by the addition of phenylalanine and tyrosine. Further, tryptophan-induced growth inhibition is exacerbated by *aexB* deletion and can be rescued by complementation. Taken together, we suggest AexB is an aromatic amino acid exporter that functions as a safety valve to prevent toxic aromatic amino acid accumulation during environmental elevation.

AexB is the first identified aromatic amino acid exporter in a gram-positive bacterium. The only other known bacterial aromatic amino acid exporter is YddG in *Escherichia coli*. While both AexB and YddG transport tryptophan, tyrosine, and phenylalanine, there is little structural homology predicted between the two proteins (45). Comparing the crystal structure for YddG to the AlphaFold predicted structure for a monomer of AexB using the RSCB PDB yields an RMSD of 5.83, indicating significant structural differences. Further, YddG belongs to the drug/metabolite transporter superfamily, while AexB belongs to a structural family of six uncharacterized proteins in *B. subtilis* (46). Thus, our study could establish a precedent for uncovering the function of these novel membrane proteins. These studies may also inform how the PII-like domain contributes to efficient efflux activity in our model. While the PII-like cytoplasmic cap of AexB contributes to the efficiency of transport, the mechanism is unclear. Perhaps the cap domain binds aromatic amino acids, increasing their local concentration for more efficient export. Further studies should investigate direct interaction between the PII-like cap domain of AexB and aromatic amino acids.

Previously characterized amino acid exporters have a range of substrates, including deleterious molecules (8, 10, 13, 14, 19, 23). Here, it is shown that AexB can export the growth-inhibiting amino acid analogs 5FT, meta-tyrosine, and 4-fluorophenylalanine. While 5FT and 4-fluorophenylalanine do not occur naturally, meta-tyrosine is exuded into the rhizosphere by fine fescue grasses in large quantities to inhibit the growth of neighboring plants (47). Perhaps AexB has been evolutionarily maintained by *B. subtilis* to cope with environmental levels of meta-tyrosine. Other organisms in the rhizosphere also produce growth inhibitory molecules which may be worth investigating as further substrates for AexB. For example, *Streptomyces griseus*, which was first isolated from soil, produces the tryptophan-derived antibiotic, indolmycin (48). Interestingly, *B. subtilis* has a 16.5-fold higher minimal inhibitory concentration to indolmycin compared to *Staphylococcus aureus* (49). Perhaps *B. subtilis* uses AexB as a defense mechanism to export such toxins or even produces its own yet uncharacterized antibiotic which is exported through AexB. Other substrates that should be investigated include amino acid synthesis intermediates. Accumulation of the tryptophan intermediate, anthranilate, has been shown to be inhibitory via participation in side reactions (50). Moreover, the buildup of tryptophan intermediates in *E. coli* has been shown to activate an efflux system (51). A broader investigation into the substrates that AexB can export is warranted and could provide further insights into its functional relevance.

It remains possible that AexB evolved to export the canonical aromatic amino acids. In support of this hypothesis is the observation that high levels of tryptophan are detrimental to *B. subtilis* growth. To our knowledge, this is the first report of tryptophan toxicity in *B. subtilis*. Perhaps AexB functions as an acute safety valve to manage momentarily elevated levels of tryptophan. In fact, there is precedent for these exporters to function as a safety valve, such as AlaE, which was shown to prevent toxic accumulation of alanine (52). In addition to regulation via TRAP, tryptophan biosynthesis is subject to feedback inhibition and can be shut down rapidly via allosteric binding of tryptophan to TrpE (53). However, TRAP-dependent inhibition of TrpP synthesis appears to be the only mechanism of regulation for tryptophan import, and fully synthesized tryptophan importers would remain active until targeted for degradation (38). Therefore, it is conceivable that AexB could act as an acute safety valve to maintain homeostasis if environmental tryptophan levels become momentarily elevated. While homeostasis is a plausible explanation for the function of AexB, it is not clear when *B. subtilis* would encounter such high levels of tryptophan in the environment.

It should be noted that AexB expression is consistently low across many tested conditions, similar to previously identified amino acid exporters AzlCD and AexA (54). Interestingly, β-alanine stress was shown to result in suppressor mutations that activate the transcription factor AerA, ultimately resulting in increased expression of AexA, which exports β-alanine allowing growth (10). It is tempting to speculate that AexB may be activated by a similar mechanism; however, we have no evidence of such. In fact, we do not observe any spontaneous increase in growth rate during WT growth in high tryptophan, suggesting that suppressor mutations are not acquired. Further, while AexA can be activated by its substrate 2,3-diaminopropionic acid, AexB is not activated by non-inhibitory levels of tryptophan (data not shown). Future studies should investigate conditions and potential mechanisms that may activate AexB.

The mechanism of tryptophan toxicity remains unclear; however, there is precedent for amino acid toxicity when intracellular levels become elevated. In *Saccharomyces cerevisiae*, it has been shown that each canonical amino acid can become growth inhibitory at concentrations of just 3–5 mM (55, 56). One possible explanation could be feedback inhibition of enzymes. In *Synechocystis* sp. phenylalanine has been shown to allosterically inhibit 3-deoxy-d-arabinoheptulosonate 7-phosphate synthase, an early enzyme in the aromatic amino acid biosynthetic pathway. This feedback inhibition ultimately results in reduced synthesis of the other aromatic amino acids, tyrosine and tryptophan, reducing the growth rate (34). Our data agree with the idea that tryptophan participates in a similar allosteric feedback inhibition in *B. subtilis*. Further experiments should investigate the intracellular levels of other amino acids in this context and explore the allosteric interaction between tryptophan and aromatic amino acid synthesis enzymes.

It is well appreciated that maintenance of amino acid concentrations is imperative for protein synthesis. As such, a holistic understanding of amino acid synthesis and transport serves to inform our understanding of basic biological systems. Perhaps less appreciated, however, are the non-proteogenic roles of amino acids. Methionine is converted to S-adenosylmethionine, which is used for methylation of DNA/RNA (57), cysteine and valine are converted to beta-lactam antibiotics (58), and various amino acids are converted to cell wall and membrane structural components such as branched-chain fatty acids, teichoic acids, and peptidoglycan linkers (59–61). These non-proteogenic roles are diverse and numerous. Tryptophan is particularly noteworthy as it serves as the precursor molecule to both serotonin and kynurenine. The serotonin and kynurenine pathways have far-reaching implications for health and disease. Serotonin functions as a neurotransmitter in higher organisms where it regulates mood and sleep but also acts as an important signaling molecule in the gut, where it regulates peristalsis and secretion of digestive enzymes. Kynurenine and its related metabolites have immunomodulatory effects, such as suppressing T cell activity, and can have both neuroprotective and neurotoxic effects. As such, tryptophan metabolism has been implicated in cancer, neurodegenerative diseases, IBD/IBS, chronic inflammation, and autoimmune disorders (62–74).

Recently, it has become appreciated that microbiota can modulate levels of host metabolite synthesis in the gut, blood, and the brain via production and synthesis of their own metabolites (75–78). As such, tryptophan metabolism is a blossoming area of study within the context of the microbiome. An analysis of over 8,000 bacterial genomes found ~40% contained tryptophan catabolic pathways, many of which belong to gut-associated phyla including Actinobacteria, Firmicutes, Proteobacteria, Bacteroidetes, and Fusobacteria (79). The prevalence of these tryptophan catabolic pathways in microbiome species, how secretion of other metabolites such as short-chain fatty acids impacts host tryptophan metabolism and vice versa, and to what extent these interactions contribute to disease are active areas of interest. It may also be interesting to consider how increased tryptophan availability due to secretion from bacteria may affect these metabolic pathways in host cells. As such, the identification of AexB as an aromatic amino acid exporter may guide the identification of other exporters in microbiome-associated species.

## MATERIALS AND METHODS

### Strains, media, and growth conditions

Two *B. subtilis* strains were used throughout this study, NCIB 3610 and 168. Bacteria were grown in lysogeny broth (LB) and seeded into MSgg-defined minimal media for growth experiments. MSgg contains 100 mM MOPS (pH 7), 5 mM potassium-phosphate buffer (pH 7), 2 mM MgCl_2_, 700 µM CaCl2, 50 µM MnCl_2_, 100 µM FeCl_3_, 1 µM ZnCl_2_, 2 µM thiamine HCl, 0.5% (vol/vol) glycerol, and 0.5% (wt/vol) monosodium glutamate. Strains were grown in LB until the stationary phase (~4.5 h), spun down at 3,000 × *g* for 3 min and washed with PBS, twice before resuspending in PBS for seeding. Five microliters of washed cells were seeded into 195 µL MSgg in 96-well microplates (Corning 3904). For co-culture experiments, 5 µL of each strain was inoculated into 190 µL of media, and for single strains, 10 µL of culture was used. Cultures were grown in the BioTek Synergy Neo2 plate reader shaking orbitally at 280 rpm at 37°C.

### DNA manipulation and transformation

All plasmid assemblies were performed using Gibson Assembly using the Gibson Assembly Master Mix (NEB). Transformation of *E. coli* and plasmid DNA extraction were performed using standard procedures (80). *B. subtilis* was transformed with plasmids using a natural competence protocol previously described and plated on LB agar with appropriate selection (81). Genetic complements with native promoters were amplified from the native NCIB genome with 1,000 bp of the native 5′ region and added to the integration vector pBS1E with MLS resistance from *E. coli* strain ECE730 (https://bgsc.org/getdetail.php?bgscid=ECE730) (82). Gene overexpression was amplified the same way as genetic complements using pBS1E, but the native promoter was swapped with pRpsD for constitutive expression.

### Construction of mutant strains by transduction

*B. subtilis* 168 mutants were obtained from a previously generated genome-scale deletion library (83). Mutations were transferred from the 168 strains to NCIB 3610 via SPP1-mediated generalized transduction (84).

### Optical density, fluorescence, and cell density measurements

Optical density was measured at 600 nm to monitor bacterial growth, and YFP fluorescence for co-culture experiments was measured with excitation/emission of 500/541 nm using the BioTek Synergy Neo2 plate reader.

### Metabolite relative quantification by LC-MS

Strains to be harvested for metabolomics were grown for 10 h in MSgg in 96-well format as described earlier. Cultures were harvested by pooling four replicates into a single microcentrifuge tube to ensure sufficient material. This pooled sample was treated as one replicate during LC-MS data collection and analysis. Pooled samples were spun down at 20,000 × *g* for 15 min at 4°C. Supernatant was transferred into a fresh microcentrifuge tube by pipette and mixed thoroughly by vortexing. Then 200 µL of supernatant was mixed with 800 µL of pre-chilled 100% MeOH and stored at −80°C overnight before submission to the metabolomics core at Northwestern University.

Samples were analyzed by high-performance liquid chromatography and high-resolution mass spectrometry and tandem mass spectrometry (HPLC-MS/MS) (85, 86). Specifically, the system consisted of a Thermo Q-Exactive in line with an electrospray source and an Ultimate3000 (Thermo) series HPLC consisting of a binary pump, degasser, and auto-sampler outfitted with an Xbridge Amide column (Waters; dimensions of 3.0 mm × 100 mm and a 3.5 µm particle size). The mobile phase A contained 95% (vol/vol) water, 5% (vol/vol) acetonitrile, 10 mM ammonium hydroxide, 10 mM ammonium acetate, pH = 9.0; B was 100% acetonitrile. The gradient was as follows: 0 min, 15% A; 2.5 min, 30% A; 7 min, 43% A; 16 min, 62% A; 16.1–18 min, 75% A; and 18–25 min, 15% A with a flow rate of 150 µL/min. The capillary of the ESI source was set to 275°C, with sheath gas at 35 arbitrary units, auxiliary gas at 5 arbitrary units, and the spray voltage at 4.0 kV. In positive/negative polarity switching mode, an *m/z* scan range from 60 to 900 was chosen, and MS1 data were collected at a resolution of 70,000. The automatic gain control (AGC) target was set at 1 × 10^6^, and the maximum injection time was 200 ms. The top five precursor ions were subsequently fragmented, in a data-dependent manner, using the higher-energy collisional dissociation (HCD) cell set to 30% normalized collision energy in MS2 at a resolution power of 17,500. Besides matching *m/z*, metabolites are identified by matching either retention time with analytical standards and/or MS2 fragmentation pattern. Data acquisition and analysis were carried out by Xcalibur 4.1 software and Tracefinder 4.1 software, respectively (both from Thermo Fisher Scientific).

### Statistical analyses

Statistical tests were calculated in GraphPad Prism 9.0. For comparisons between two independent groups, a Student’s *t*-test was used. For multiple groups, a one-way ANOVA was performed, followed by post hoc multiple comparisons. Significance was accepted at *P* < 0.05. The details of the statistical tests carried out are indicated in the respective figure legends.

## SUPPLEMENTAL MATERIAL

Supplemental Material mbio.00231-26-s0001.docx Fig. S1 to S8.

ASM does not own the copyrights to Supplemental Material that may be linked to, or accessed through, an article. The authors have granted ASM a non-exclusive, world-wide license to publish the Supplemental Material files. Please contact the corresponding author directly for reuse.
