# Supplementary material for: AexB is an aromatic amino acid exporter that functions as a metabolic safety valve
Source: mBio. 2026 Apr 3;17(5):e00231-26. doi: 10.1128/mbio.00231-26 (PMC13170310; doi:10.1128/mbio.00231-26)

**SUPPLEMETNAL MATERIAL**

**Supplemental Figure 1 – Transposon insertion site for *aexB* overexpression**

Sequence information for the precise insertion site of the TnFlx transposon in the 27 identified *aexB* overexpression hits resistant to 5FT.

**
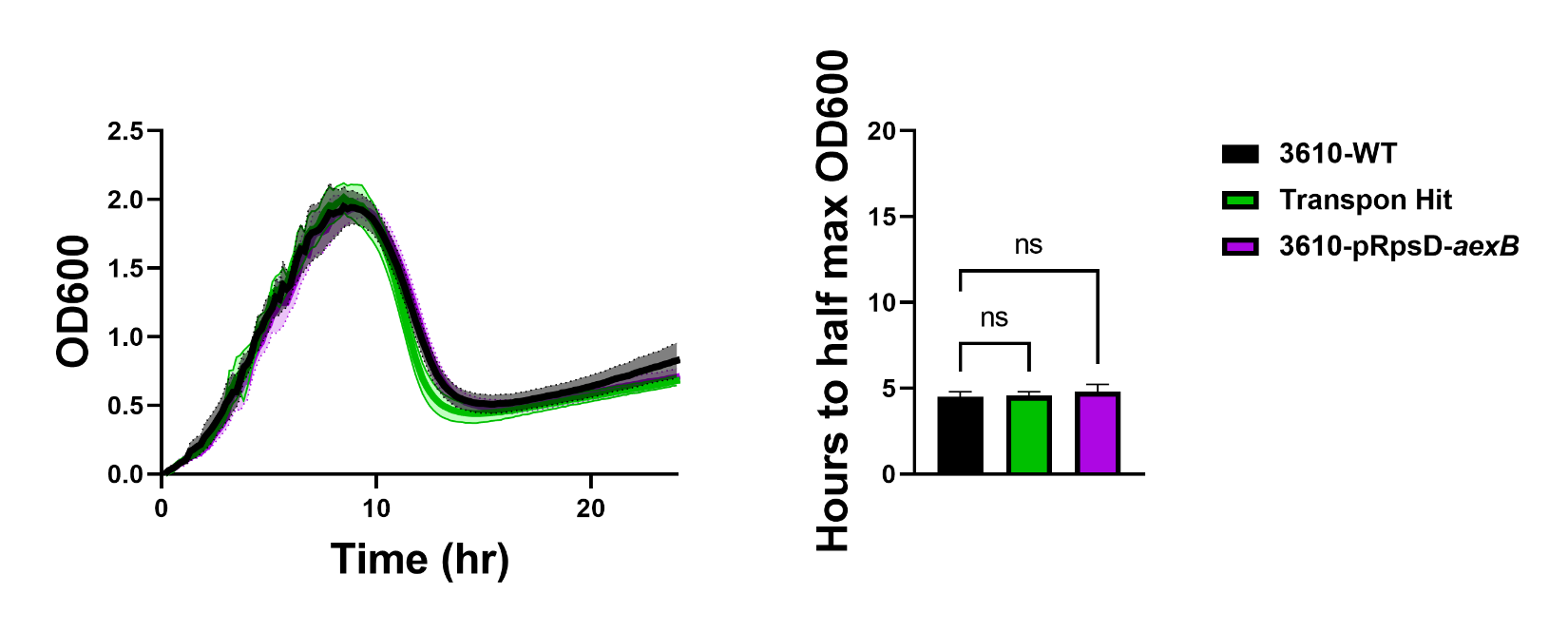

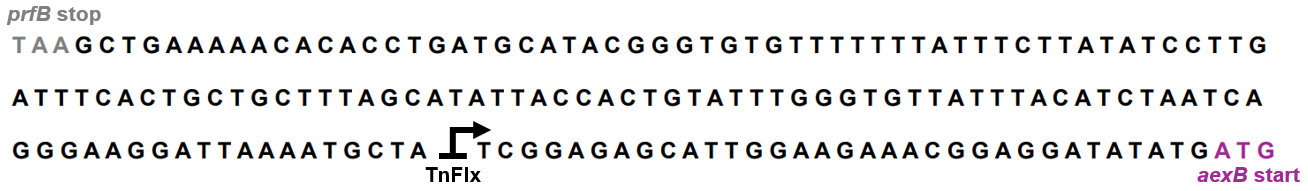
**

**Supplemental Figure 2 – No growth defect is associated with the transposon hit nor overexpression of *aexB***

Average growth curves in 96-well plate reader format for 3610-WT, a representative transposon hit and 3610-pRpsD-*aexB* in MSgg (left) and quantification of the time it took for each strain to reach half the maximum OD600 (right). Shaded area is SEM. Statistical significance was determined by one-way ANOVA followed by post-hoc multiple comparisons. *n=12*

**
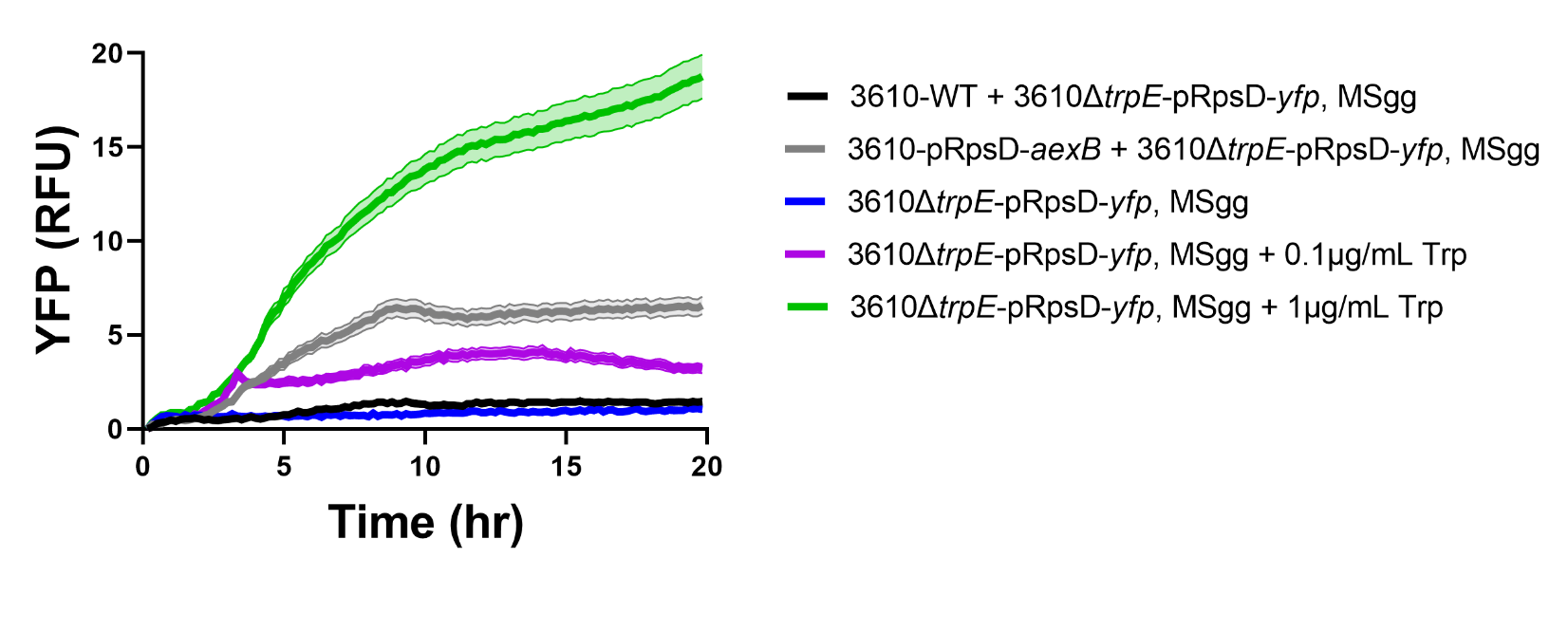
**

**Supplemental Figure 3 – Overexpression of *aexB* results in export of tryptophan in the low micromolar range.**

Average YFP signal in 96-well plate reader format for a tryptophan auxotroph expressing a constitutive YFP reporter, 3610∆*trpE*-pRpsD-*yfp*, co-cultured with either 3610-WT or 3610-pRpsD-*aexB* in MSgg, as well as tryptophan dose response in MSgg for 3610∆*trpE*-pRpsD-*yfp* alone. Shaded area is SEM. *n=12*

**Supplemental Figure 4 – 3610 can more efficiently transport tryptophan than 168 via an unknown mechanism**

(A) Average growth curves in 96-well plate reader format for 168-WT and 168∆*trpP* and (B) 3610-WT and 3610∆*trpE*,∆*trpP* in MSgg supplemented with 50µg/mL trp. Shaded area is SEM. *n=6* and *n=5*, respectively.

**
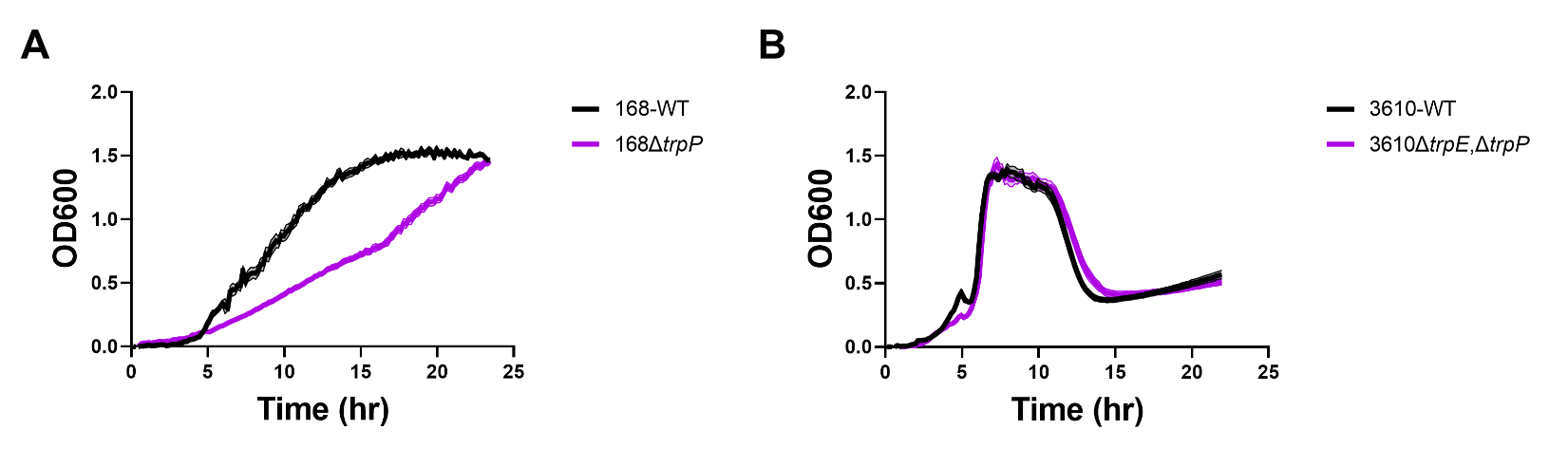
**

**
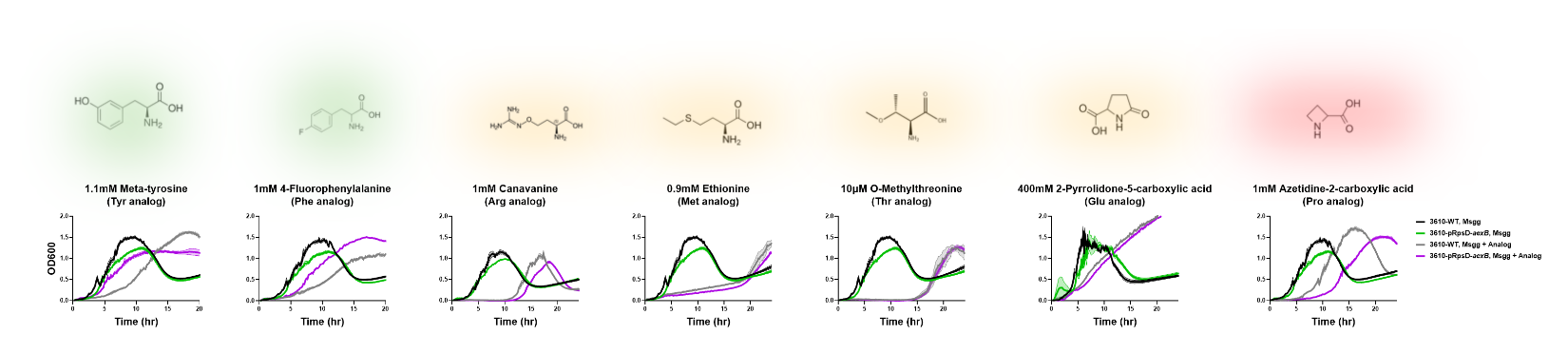
**

**Supplemental Figure 5 – Screening *aexB* overexpression growth on various amino acid analogs suggest AexB exports aromatic amino acids but not non-aromatic amino acids**

Panel of average growth curves in 96-well plate reader format for 3610-WT and 3610-pRpsD-*aexB* in MSgg or MSgg supplemented with various amino acid analogs. Shaded area is SEM. *n=4*

**
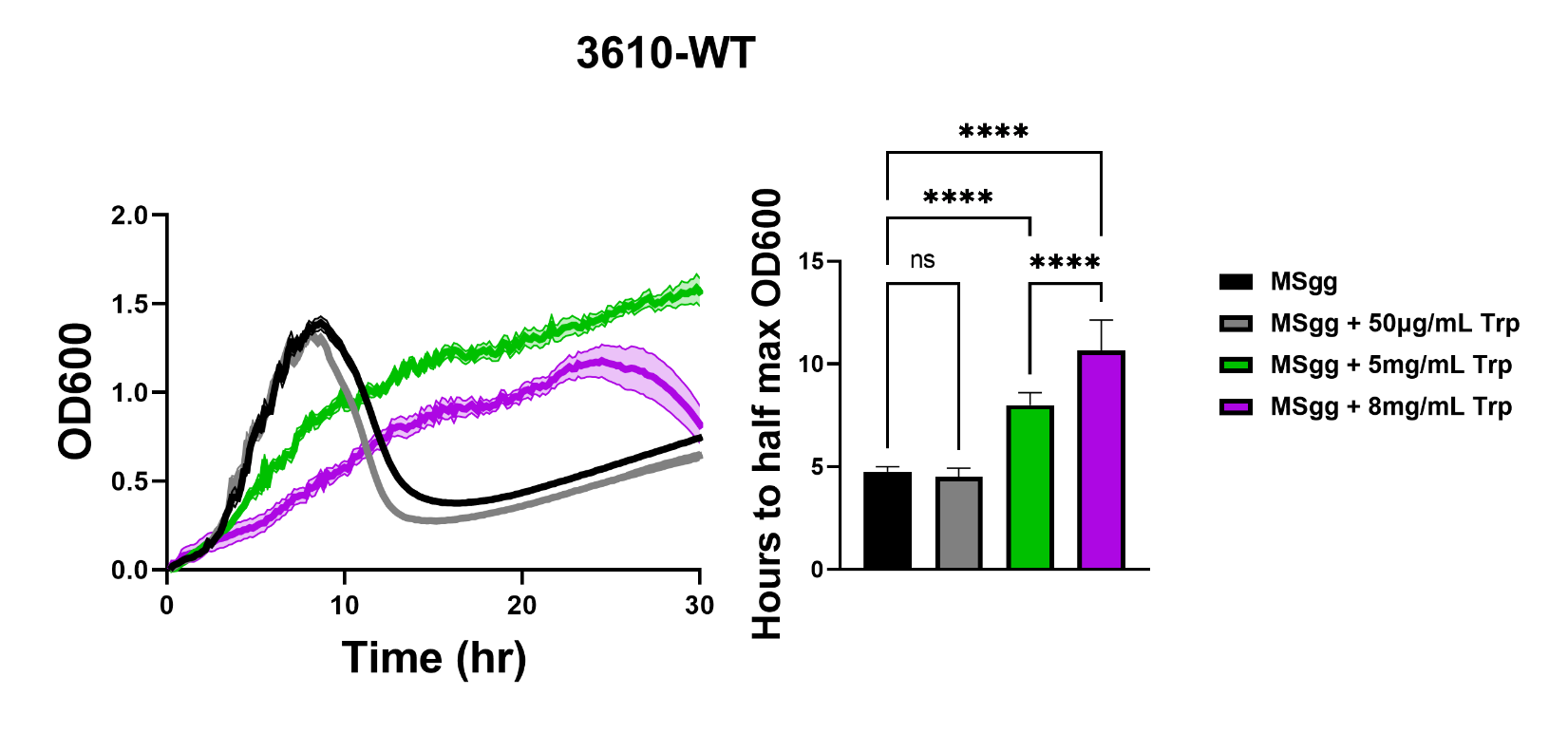
**

**Supplemental Figure 6 – Exogenously supplied tryptophan is growth inhibitory in a dose dependent manner**

(A) Average growth curves in 96-well plate reader format for 3610-WT in MSgg with varying levels of trp (left) and quantification of the average time it took to reach half the maximum OD600 in each condition. Shaded area is SEM. Statistical significance was determined by one-way ANOVA followed by post-hoc multiple comparisons. *n=8, ****P<0.0001*

**
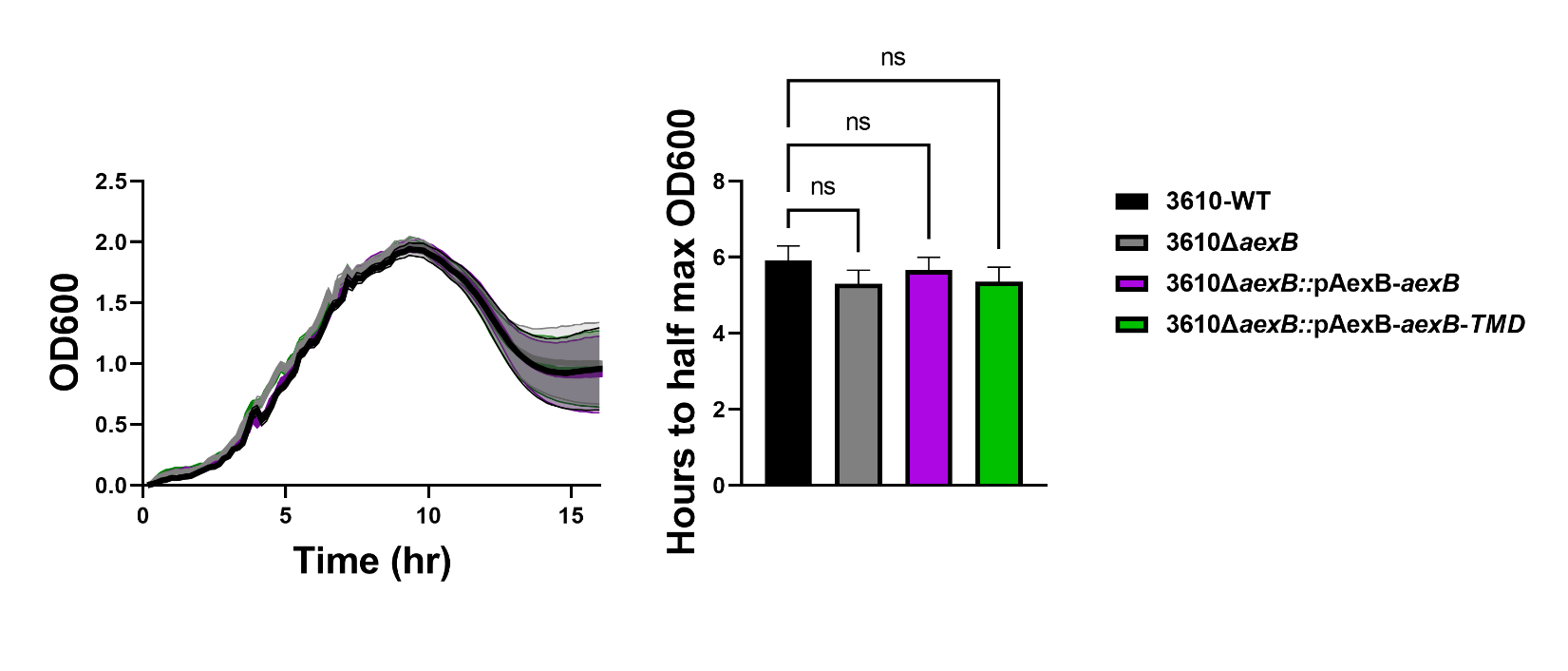
**

**Supplemental Figure 7 – No growth defect is associated with AexB deletion, complementation nor transmembrane domain complementation**

Average growth curves in 96-well plate reader format for various strains in MSgg (left) and quantification of the average time it took each strain to reach half the maximum OD600 (right). Shaded area is SEM. Statistical significance was determined by one-way ANOVA followed by post-hoc multiple comparisons. *n=12*

**Supplemental Figure 8 – AexB cap domain contributes to efficient export of tryptophan**

Average growth curves in 96-well plate reader format for various 168 strains in MSgg + 1µg/mL Trp (left) and quantification of the average time it took each strain to reach half the maximum OD600 (right). Statistical significance was determined by one-way ANOVA followed by post-hoc multiple comparisons. *n=12, ***P= 0.0001*


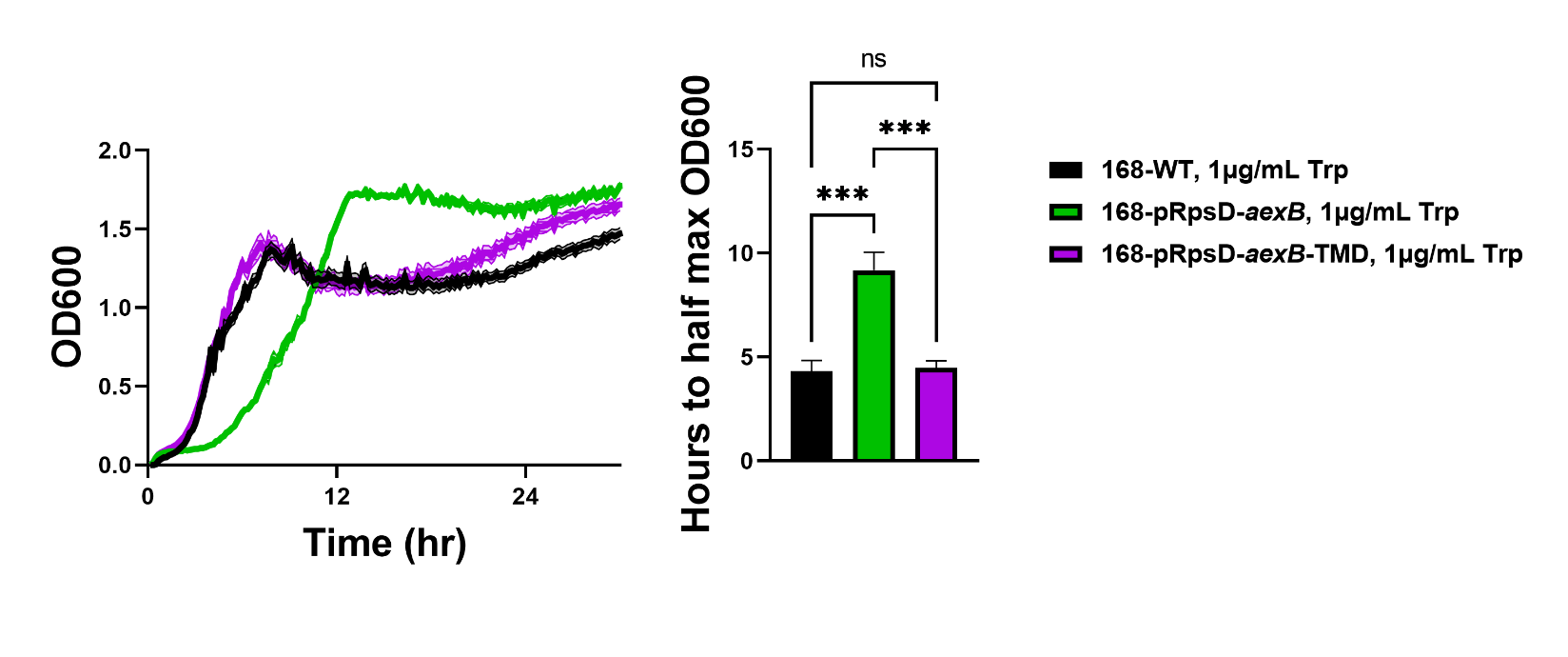

Supplement: Supplemental Material — Fig. S1 to S8. [file mbio.00231-26-s0001.docx]
